# Prioritizing core components of successful transitions from child to adult mental health care**:** a national Delphi survey with youth, caregivers, and health professionals

**DOI:** 10.1007/s00787-021-01806-6

**Published:** 2021-06-05

**Authors:** Kristin Cleverley, Emma McCann, David O’Brien, Julia Davies, Kathryn Bennett, Sarah Brennenstuhl, Lynn Courey, Joanna Henderson, Lianne Jeffs, Joshua Miller, Tony Pignatiello, Jessica Rong, Emily Rowland, Katye Stevens, Peter Szatmari

**Affiliations:** 1 Lawrence S. Bloomberg Faculty of Nursing, University of Toronto, 155 College Street, Suite 130, Toronto, ON M5T 1P8 Canada; 2 Yorktown Family Services, Toronto, Canada; 3 Department of Health Research Methods, Evidence, and Impact, Faculty of Health Sciences, McMaster University, Hamilton, Canada; 4 Sashbear Foundation, Toronto, Canada; 5 Margaret and Wallace McCain Centre for Child, Youth and Family Mental Health and Campbell Family Mental Health Research Institute, Centre for Addiction and Mental Health, Toronto, Canada; 6 Department of Psychiatry, Faculty of Medicine, University of Toronto, Toronto, Canada; 7 Lunenfeld-Tanenbaum Research Institute, Sinai Health, System, Toronto, Canada; 8 Institute of Health Policy, Management and Evaluation, University of Toronto, Toronto, Canada; 9 Li Ka Shing Knowledge Institute, St Michael’s Hospital, Toronto, Canada; 10 Factor-Inwentash Faculty of Social Work, University of Toronto, Toronto, Canada; 11 Department of Psychiatry, The Hospital for Sick Children, Toronto, Canada; 12 Centre for Brain and Mental Health and Department of Psychiatry, The Hospital for Sick Children, Toronto, Canada; 13 The Cundill Centre for Child and Youth Depression, Centre for Addiction and Mental Health, Toronto, Canada

**Keywords:** Transition, Continuity of care, Mental health, Youth, Delphi study, Consensus, Patient-oriented research

## Abstract

Youth accessing mental health care often experience a disruption in care as they attempt to transition between child and adolescent mental health services (CAMHS) and adult mental health services (AMHS). Few studies have evaluated interventions seeking to improve the experience and outcomes of CAMHS–AMHS transitions, in part due to lack of consensus on what constitutes best practices in intervention success. As such, the aim of this study was to engage patients, caregivers, and clinicians to prioritize core components of successful CAMHS–AMHS transitions which can be used in the design or evaluation of transition interventions. As such, a Delphi study was conducted to determine core components of successful CAMHS–AMHS transitions. Guided by the principles of patient-oriented research, three balanced expert panels consisting of youth, caregivers, and clinicians ranked and provided feedback on the importance and feasibility of core components of CAMHS-AMHS transitions. Components endorsed as feasible or important with ≥ 70% agreement from any panel moved to the next round. As a result, a list of 26 core components of CAMHS–AMHS transitions has been refined which can be used in the design, implementation, or evaluation of interventions intended to improve transition experiences and outcomes for youth in mental health care. Youth and families were engaged in an expert advisory role throughout the research process, contributing their important perspectives to the design and implementation of this study, as well as interpretation of the findings.

**Supplementary Information:**

The online version contains supplementary material available at 10.1007/s00787-021-01806-6.

## Introduction

Mental illness is a prevalent issue, particularly among youth. Approximately 1 in 5 children and adolescents have at least one mental health problem [1, 2]; with up to 70% persisting into adulthood [3]. Adolescents and young adults are also increasingly accessing care for their mental health in recent years [4]. For those using mental health services, the transition of care from child and adolescent mental health services (CAMHS) to adult mental health services (AMHS) is acknowledged as a challenging health systems hurdle [5–8]. A common point of termination in mental health services occurs at age 18, during which youth receiving CAMHS must discontinue care and transition out [9, 10]. A successful transition ensures Continuity of Care (CoC) through a planned health care process that addresses personalized therapeutic and developmental needs of the patient [11]. If CoC is not achieved at the point of transition, youth who are engaged in CAMHS and require ongoing mental health care after age 18 are at risk of worsening mental health and functioning [12–14]. Currently, as many as 6 of 10 youth disengage from service use [12, 15]. Negative outcomes of disengaging from mental health care services include more severe mental health problems, inadequate medication monitoring, re-engaging later in crisis, increased involvement with the justice system, among others [12, 16–19]. Despite this, there is a lack of research evaluating interventions to improve CoC for youth transitioning from CAMHS to AMHS and a lack of agreement on what interventions designed to support CoC during transitions should focus on.

A scoping review of interventions to support CAMHS–AMHS transitions revealed that few interventions had been evaluated [20]. This review concluded that research may be impeded by the lack of psychometrically sound instruments to appropriately evaluate transition programs and interventions. In our own scoping review of components of CAMHS to AMHS interventions, we identified and extracted an extensive list of 26 core components from 57 articles and 33 documents [21]. Results demonstrated a lack of consensus among experts as to which components are important and feasible to implement in the evaluation of programs to improve CoC from child to adult mental health services [21]. Recent work to identify core process and outcome indicators for child to adult care transitions in other health care specialties, such as hematology and rheumatology, have primarily engaged health care professionals to the exclusion of youth and families [22–25]. Within mental health care, however, research has demonstrated that various stakeholders (i.e. youth, caregivers, and clinicians) have different experiences and needs when it comes to supporting transitions in mental health care [17, 26]. Furthermore, youth and caregivers have demanded to not only be included in this research but also to be engaged in its co-design [27, 28]. As such, studies ought to engage patients (youth and caregivers) as experts alongside clinicians, policy-makers and researchers when developing tools for evaluating transitions from CAMHS to AMHS. Delphi studies are an ideal methodology for integrating diverse stakeholders in a decision-making process.

A Delphi study is a multi-staged survey designed to achieve consensus among experts on an important issue [29, 30] and has been used extensively in health care [31] and more recently mental health care [32]. Information is gathered using a series of online survey rounds with feedback from the previous round integrated into the subsequent rounds using clear and transparent criteria. The Delphi method prevents domination of the consensus process by one or a few experts by including individuals from various locations and areas of expertise to respond anonymously [33]. This facilitates voices from all stakeholders to be heard equally. This method is particularly effective in engaging stakeholders, including patients and clinicians, in research and ensuring that the priorities of all participants (including youth and their caregivers) are known [30, 34, 35].

With this in mind, the objective of this Delphi study was to engage patients, caregivers, and clinicians as experts in prioritizing and refining the core components of effective CAMHS–AMHS transitions that were identified in our scoping review [21].

## Methods

### Study design

A modified e-Delphi method was utilized. This method involved creating initial survey items based on an extensive review of the available literature, as well as input from expert advisors with lived experience [36–40]. Survey items were then distributed to three expert panels for ranking based on importance and feasibility. This study was reviewed by the University of Toronto Health Sciences and the Centre for Addiction and Mental Health Research Ethics Boards.

### Expert advisory committee

This study utilized the priniciple of Patient-Oriented Research (POR), guided by the Strategy for Patient-Oriented Research Patient Engagement Framework (SPOR-PEF) in developing the methodology needed to enable patients to engage as experts [41]. As such, a group of patients and knowledge users (KU) formed a study Expert Advisory Committee (EAC). The EAC was composed of three youth, two caregivers, and one sibling, all with lived experience [42–44], as well as four knowledge users [45] (one health care administrator, one decision-maker, and two community leaders) from relevant child and youth mental health agencies. The EAC met regularly (every 2–4 months) with the study team over the course of the project to advise on recruitment strategies, development of the supplemental materials (i.e. informational video [46]) interpretation of the findings, knowledge translation goals and strategies to reach youth, families and other stakeholders, and dissemination of the project findings.

### Delphi expert panel members

Participants who met criteria for one of the three expert panel groups were recruited. These panels include (a) Panel 1, comprised of service users, specifically youth (aged 19–25) who have received mental health care from a CAMHS and have transitioned to an AMHS; (b) Panel 2, comprised of family members or caregivers of youth aged 19–25 who had received mental health care from a CAMHS and have transitioned to AMHS at the time of recruitment; and (c) Panel 3, comprised of clinicians from any professional designation (i.e. registered nurse, physician, etc.), who have had at minimum one year of experience in research or clinical treatment of youth and/or early adult mental health care (ages 16–25) and mental health administrators who have responsibility for the delivery and/or oversight of CAMHS.

### Recruitment of expert panel members

Purposive sampling recruitment took place over a period of 6 weeks. For Panel 1 (youth) and 2 (caregivers) our project partners and EAC members sent out the project information via email to their contacts and listserv’s, which included youth, caregivers, and/or clinician/administrator. For Panel 3, we utilized three strategies to identify potential panel members: (1) relevant conference attendee lists were searched, (2) professional associations membership lists were reviewed, and (3) authors of manuscripts that were reviewed in the scoping review were search. All identified experts were then invited via email to participate in the survey. If interested, individuals contacted the research team via email at which point they were sent an invitation email by the principal investigator (KC) to introduce the study and confirm their eligibility. Stakeholders and experts were given the opportunity to recommend additional individuals they felt should be included as participants. In recognition of their time and commitment, youth and caregiver participants received a $40 honorarium gift card for participating in Round 1 of the Delphi survey. In Round 2 all participants (including clinicians and administrators) across all panels received the honorarium for participation to optimize response rate and at the suggestion of the study EAC.

### Survey development

A scoping review of the literature was previously conducted by the research team, which informed the items that would be included in the Delphi study [21]. In total, 26 core components, grouped into six core elements, of CAMHS to AMHS transitions were derived from the academic and gray literature in this scoping review (see column “Initial description of core components” in Table 1). This list was reviewed in-person by the EAC for modifications and edits (e.g. wording, order, omissions) before the first Delphi survey round was administered to participants. Following their review, the EAC determined, by consensus, that a core component regarding access to peer support should be included, leading to the addition of component 4.11. The final survey consisted of 27 core components that were rated on a 9-point Likert scale on two domains: importance and feasibility. Importance referred to how significant or meaningful the core component is to the measurement of transition quality between CAMHS and AMHS. Feasibility referred to how realistic it would be to implement the core component in practice. These criteria have previously been used in Delphi studies examining transitions to adult healthcare services [22, 25], and their definitions are aligned with prior Delphi studies [47, 48]. All three panels of participants were provided space for additional comments and/or feedback regarding each core component, if desired. At the end of the survey, participants could also provide overall comments, including suggestions of any missing components that should be added.

**Table 1:** Initial components of successful CAMHS-AMHS transitions generated by scoping review by Cleverley et al. (2020) and final components following Delphi rounds

Component	Initial description of component	Final description of component
Core Element 1.0 Transition Policy		
1.1	Develop an integrated care pathway that describes the steps that make up the transition process	Develop an integrated care pathway that describes the steps that make up the transition process
1.2	Develop a transition policy/statement with input from youth and caregiver(s) that describes the program/agencies approach to transitions	Develop an organization-specific transition policy with youth (with input from family members/caregivers) that describes the organization’s approach to mental health care transitions, and make it publicly available
1.3	Develop a needs-led and developmentally appropriate transition protocol that is agreed upon by both CAMHS and AMHS, which includes standards for communication, information sharing and record-keeping	Develop a youth-centered and developmentally appropriate transition protocol in collaboration with both the child and adolescent mental health services and the adult mental health services, that outlines standards for communication and information sharing
1.4	Ensure that all staff have the knowledge, skills and training to effectively support the agency/services approach to transitions	Ensure that all staff have the knowledge, skills and training to effectively support the agency/services approach to transitions
1.5	Determine a clear role for all individuals (youth, caregivers, CAMHS and AMHS staff) involved in the transition process	Determine a clear role for all individuals (e.g. youth, child and adolescent mental health services and adult mental health services staff, peer support workers, transition support workers and family members/caregivers if appropriate) involved in the transition of care, informed by the needs of each youth
1.6	Include young person and their caregiver(s) at all phases of transition and decision-making	Partner with the youth (and family members/caregivers, if appropriate) at all phases of transition and decision-making
1.7	Establish the process for evaluation and assessment of transition protocol	Establish a plan to evaluate the organization’s transition protocol
Core Element 2.0 Transition Tracking and Monitoring		
2.1	Establish criteria and process for identifying youth who will be transitioning in and out of the agency/service	Establish organization-specific criteria and process for identifying youth who will be transitioning out of child and adolescent mental health services
2.2	Establish a transition flow sheet or log book to track youth who transition between CAMHS and AMHS	Establish a transition flow sheet or log book that tracks the completion of important steps as youth transition out of child and adolescent mental health services
Core Element 3.0 Transition Readiness		
3.1	Conduct regular transition readiness assessments, and in collaboration with youth and their caregiver(s), identify needs and goals, update regularly	Conduct regular transition readiness assessments, and in collaboration with youth (and family members/caregivers, if appropriate) identify youths’ needs and goals, update regularly
3.2	Educate youth and their caregiver(s) about differences in CAMHS and AMHS programs and services	Provide youth (and their family members/caregivers, if appropriate) information about what to expect from adult mental health services
3.3	Develop individualized transition plan in collaboration with youth and their caregiver(s) at least 6 months before planned transition	Develop individualized transition plan in collaboration with youth (and their family members/caregivers, if appropriate) a minimum of 6-months before planned transition, or as early as possible
Core Element 4.0 Transition Planning		
4.1	Identify all stakeholders involved in the transition	Identify everyone involved in the transition (e.g. child and adolescent mental health services, adult mental health services, youth and family members/caregivers, transition workers, primary care practitioners, etc.)
4.2	In collaboration with youth and their caregiver(s), identify a provider (AMHS)	Collaborate with youth (and family members/caregivers, if invited by youth) to identify adult services that are an appropriate fit
4.3	Confirm the AMHS agency/service eligibility criteria	Confirm the adult mental health service eligibility criteria
4.4	Discuss optimal timing of transfer with youth and caregiver(s)	Agree on optimal timing of transfer with youth and other relevant service providers in the circle of care (and family members/caregivers, if appropriate)
4.5	In collaboration with youth and their caregiver(s), complete and regularly update the individualized transition plan (including, for example: readiness assessment findings, goals and prioritized actions, medical summary, emergency care plan)	In collaboration with youth (and their family members/caregivers, if appropriate), complete the individualized transition plan and keep it up-to-date (including, for example: readiness assessment findings, goals and prioritized actions, clinical summary, crisis plan)
4.6	Identify the most responsible clinician (i.e. transition/key worker) to ensure continuity in relationship and contact person during the transfer of care	Identify the most responsible person (i.e. child and adolescent mental health services clinician, transition worker) to coordinate the transition process, act as the main contact, and ensure continuity in the youth’s care
4.7	At least 6 months prior to transfer of care CAMHS clinician initiate transition planning with the AMHS provider, including holding joint working meetings or a period of parallel care; include youth and their caregiver(s) in meetings	At least 6-months prior to transfer of care child and adolescent mental health services clinician initiate transition planning with the adult mental health services provider, which may include joint working meetings or a period of parallel care; include youth (and their family members/caregivers, if appropriate) in meetings
4.8	Develop communication processes with primary care provider (i.e. family physician, nurse practitioner) to ensure they have consistent up-to-date medication and treatment information	With youth’s consent, communicate processes with primary care provider (i.e. family physician, nurse practitioner, and pharmacist) to ensure they have consistent up-to-date medication and treatment information
4.9	Provide youth and caregiver(s) with contact information for self-care management resources, culturally appropriate community supports, and community mental health resources	Provide youth and caregiver(s) with up-to-date contact information for developmentally appropriate self-care management resources, community supports, and community mental health resources
4.10	Provide community and health resources to the youth and their caregivers, in the event of withdrawal from AMHS service	Provide developmentally appropriate community and health resources to the youth (and their family members/caregivers, if appropriate), in the event that the youth does not transition to adult mental health services, withdraws from adult mental health services, or only desires episodic contact with adult mental health services
4.11	N/A	If desired by youth, facilitate connections to peer support during the transition process
Core Element 5.0 Transfer of Care		
5.1	A specific meeting or case conference should be held with all stakeholders (i.e. youth, caregivers, CAMHS and AMHS clinician) to handover care	A specific meeting or case conference should be held with everyone involved in the transition to handover care (i.e. youth, child and adolescent mental health services and adult mental health services clinician, transition workers, and family members/caregivers if appropriate)
5.2	Transfer young adult when his/her condition is stable	N/A
5.3	Complete all documents in transfer package (e.g. referral letter, individualized transition plan, medical records), send to adult practice/external agencies, and confirm receipt	In collaboration with youth, complete all documents in transfer package (e.g. referral letter, individualized transition plan, clinical records). With youth’s consent send to adult mental health services and/or primary care provider, and confirm receipt
Core Element 6.0 Transfer Completion		
6.1	Most responsible clinician contact the youth and caregiver(s) 3–6 months after last CAMHS visit to confirm transfer to AMHS and offer consultation assistance	The person most responsible for the transition, contacts the youth (and family members/caregivers, if appropriate) 3 to 6 months after last child and adolescent mental health service visit, or sooner if necessary, to confirm transfer to adult mental health services

Demographic questions specific to each expert panel were included as part of Round 1 (see Table 2). Youth, caregiver, and clinician/administrator experts were asked panel-specific demographic information, based on review from the EAC, to ensure that collected data was appropriate and provided meaningful information about each panel. For example, youth were asked to provide information such as their age, gender, and living situation, whereas clinicians were asked to provide primary clinical population served, years of professional experience, etc.

**Table 2:** Characteristics of expert panels

Panel		Round 1 N (%)	Round 2 N (%)
Clinicians and administrators		*N* = 21	*N* = 19
Primary practice location	Academic Hospital	16 (76.2)	13 (68.4)
	Community Mental Health Clinic	3 (14.3)	2 (10.5)
	Other	2 (9.5)	2 (10.5)
	Missing		2 (10.5)
Province	Ontario	17 (81.0)	13 (68.4)
	Quebec, BC, Manitoba, Nova Scotia	4 (19.0)	4 (21.1)
	Missing		2 (10.5)
Professional group	Nurse	5 (23.8)	5 (26.3)
	Psychiatrist	8 (38.1)	6 (31.6)
	Service Manager	3 (14.3)	2 (10.5)
	Other	5 (23.8)	4 (21.1)
	Missing		2 (10.5)
Primary clinical population children/youth	Yes	20 (95.2)	16 (84.2)
	Missing		2 (10.5)
Years of service in profession, mean (SD)		20.5 (10.2)	22.0 (10.7)
Years of work at current agency, mean (SD)		17.7 (9.2)	19.0 (9.7)

Caregivers		*N* = 17	*N* = 14
Province	Alberta	2 (11.8)	1 (7.1)
	Ontario	15 (88.2)	13 (92.9)
Age (caregivers), mean (SD)		53.6 (7.2)	53.9 (7.3)
Relationship to child	Biological mother	11 (64.7)	9 (64.3)
	Biological father	2 (11.8)	1 (7.1)
	Adoptive parent/other	4 (23.5)	4 (28.6)
Marital status	Single/separated/divorced	7 (41.2)	5 (35.7)
	Married/common law	10 (58.8)	9 (64.3)
Working status	Yes, full or part time	12 (70.6)	11 (78.6)
Age (child), mean (SD)		21 (1.5)	20.9 (1.6)
Child currently living with caregiver	Yes	11 (64.7)	9 (64.3)
Child's gender identity_2_	Female	10 (58.8)	9 (64.3)
Child's diagnosis_3_	Anxiety disorder	11 (64.7)	11 (78.6)
	Personality disorder	7 (41.2)	7 (50.0)
	Mood disorder	6 (35.3)	5 (35.7)
Years since first diagnosis	< 5 years	7 (43.8)	6 (46.2)
	>/= 5 years	9 (56.3)	7 (53.8)
	Missing	1 (5.9)	1 (7.1)
Currently accessing mental health care	Yes	10 (58.8)	8 (57.1)
Age child first accessed mental health care, mean (SD)		14.2 (4.4)	15.0 (3.4)

Youth		*N* = 20	*N* = 18
Province	Nova Scotia, Quebec	4 (20.0)	3 (16.7)
	Ontario	16 (80.0)	15 (83.3)
Gender identity^b^	Female	16 (80.0)	14 (77.8)
	Male	2 (10.0)	2 (11.1)
	Non-binary	2 (10.0)	2 (11.1)
Age, mean (SD)		21.5 (1.8)	21.4 (1.8)
Cultural groups most identified with	Black, Latin American, Asian	6 (30.0)	6 (33.3)
	Mixed heritage	5 (25.0)	5 (27.8)
	White	9 (45.0)	7 (38.9)
Current living situation	Own apartment/home/other	9 (45.0)	8 (44.4)
	With parent(s)/family home	11 (55.0)	10 (55.6)
Currently enrolled in school	Yes	12 (60.0)	11 (61.1)
Currently working	Yes	14 (70.0)	12 (66.7)
Diagnosis^c^	Anxiety disorder	19 (95.0)	17 (94.4)
	Personality disorder	6 (30.0)	4 (22.2)
	Mood disorder	13 (65.0)	12 (66.7)
Years since first diagnosis	< 5 years	8 (40.0)	8 (44.4)
	>/= 5 years	12 (60.0)	10 (55.6)
Currently accessing mental health services	Yes	11 (55.0)	10 (55.6)
Age first accessed mental health care, mean (SD)		14.8 (2.9)	14.7 (3.1)

^a^Female and male were the only identities reported

^b^> 1 diagnosis can be reported

### Determining consensus

Having ≥ 70% of any of the three panels highly endorse a core component (i.e. rate it at 8 or 9 on the 9-point Likert scale) on either the importance or feasibility domain ensured that the component was carried over onto the next round [49].

### Delphi rounds

Each round of the survey was developed and administered using a secure web-based software platform designed to support data capture for research studies, REDCap (Research Electronic Data Capture) [50]. Participants could complete the survey over several sittings, saving their answers and returning at a later time, if needed. Participants were invited to participate via email and provided a web link to the survey. Each round was open for 4 weeks. Participants who complete at least one core component question were considered to be partials and included in the study, even if they did not complete the entire survey. To optimize response rates, reminder emails with the survey links were sent every 7 days to participants who either did not open the survey or only partially completed the survey. Partial and complete participants from Round 1 were invited to participate in Round 2. The de-identified quantitative (panel-specific range of the ratings and mean rating for each core component) and qualitative (comments across all panels, edits to the core components, and the suggestions to add new core components) results from Round 1 were included in Round 2 [32]. In Round 2 participants were asked to review the information from Round 1 and either maintain or modify their original ratings. All data was collected between April and July 2018.

### Statistical analysis

All analyses were undertaken using SPSS 25 [51].The demographic characteristics of each panel were summarized using descriptive statistics, including frequency counts and proportions, and means and standard deviations.

For each of the 27 core components, the percentage of participants within each panel who highly endorsed each core component (i.e., rated 8 or 9) was calculated separately for importance and feasibility. Any core component highly endorsed by ≥ %70 of at least one panel on importance or feasibility was retained for the second round. This procedure was followed for both Round 1 and Round 2.

### Qualitative analysis

Qualitative comments from Round 1 were reviewed and summarized by the research team and reviewed with the EAC. Comments were selected if they were relevant and re-occurring among panel experts. Qualitative comments were extracted and analyzed using Braun and Clarke’s [52] process of thematic analysis to identify key themes of experts’ ratings. This involves familiarization with the data through extensive review, and subsequent generation of themes, which in turn need to be reviewed and defined. One graduate prepared research assistant reviewed all comments and grouped each one into overarching themes. A qualitative review after Round 2 further sub-categorized comments based on their context. The PI (KC) reviewed the preliminary analysis to ensure that the themes accurately captured the depth of the comments.

## Results

### Participant characteristics

Of the 87 experts sent the Round 1 survey, 58 participated in the survey, resulting in a participation rate of 66.7%. The three panels included 21 clinicians and administrators, 17 caregivers, and 20 youth. All participants who provided a response in Round 1 (58) were sent the Round 2 survey, of which 51(87.9%) responded to at least one item.

In the caregiver panel, nearly two-thirds were biological mothers (64.7%), and most resided in the province of Ontario, Canada (88.2%). Just over half had female children (58.5%) and their child’s first access to mental health care was, on average, at age 14 (sd = 4.4). The most commonly reported diagnosis was an anxiety disorder (64.7%), and the majority of their children were still receiving mental health care (58.8%). See Table 2 for a full description of the panels.

The youth experts were mostly female (80.0%) and from Ontario (80.0%). Just over half were currently enrolled in school (60.0%) and living with parents/in a family home (55.0%). Almost all reported a diagnosis of an anxiety disorder (95.0%), with fewer reporting a mood (65%) and/or personality (30.0%) disorder. On average, the youth first accessed mental health care at age 15 (sd = 2.9), and many were still using mental health services (55%). The mean age of the youth experts was 21.5 (sd = 1.8). See Table 2.

In the clinician and administrator panel of experts, the largest professional group was psychiatrists (38.1%), followed by nurses (23.8%) and service managers (14.3%). Most of the panel members were practicing at an Ontario academic hospital (81.0%), and nearly all primarily with a clinical population of children and youth under 18 (95.2%). On average, the panel members had worked in their profession for 20.5 years (sd = 10.2). See Table 2.

### Round 1

Results of the Round 1 survey are displayed in Supplementary File 1, indicating the number and percentage of experts ranking each core component as 8 or 9 (i.e. highly endorsed) for importance or feasibility for each panel.

*Importance* Among clinicians/administrators, the level of agreement for importance was the greatest for core component 1.6, which was highly endorsed by 100% (see Supplementary File 1). Eight other core components were highly endorsed by ≥ 90% of the panel (1.4, 1.5, 3.2, 3.3, 4.2, 4.3 4.8–4.10), while six core components did not reach ≥ 70% agreement (2.2, 3.1, 4.5, 4.11, 5.2 and 6.1). Among caregivers, the level of agreement was the greatest for core components 4.6 and 4.7, which were highly endorsed by 92.9%. These were the only two core components highly endorsed by ≥ 90% of the panel; only two core components did not reach ≥ 70% agreement (2.2 and 4.3). Among youth, level of agreement for importance was the greatest at just over 94% highly endorsed for core components 1.6 and 4.2; these were the only two core components highly endorsed by ≥ 90% of the panel. Seven core components did not reach ≥ 70% agreement (1.1, 1.3, 2.1, 4.1, 4.4, 4.6, and 5.2). Across the panels, 14 core components were highly endorsed by ≥ 70% in each group, while none the core components were endorsed by < 70% in all three panels.

*Feasibility* Within all panels, levels of agreement for feasibility were much lower. Among the clinicians/administrators, the greatest level of agreement at ≥ 70% highly endorsed was found for core components 4.3, 4.8, and 4.9, meaning that the remaining 25 core components did not reach 70% agreement (see Supplementary File 1). Among the caregivers, level of agreement was greatest at 78.6% highly endorsed for core component 4.9, with five other core components being highly endorsed by ≥ 70% (3.2, 4.1, 4.8, 4.10, and 6.1). Among the youth, the greatest level of agreement was 77.8% highly endorsed for core component 4.9, with only one other core component achieving ≥ 70% agreement (4.2). Across the panels, one core component was endorsed by ≥ 70% (4.9) in each group.

#### Changes from Round 1 to Round 2

Only 1 core component (5.2) was removed and no new core components were added, resulting in a total of 26 core components to be used for Round 2.

### Round 2

Of the experts who participated in Round 1, 19 clinicians/administrators (90.4%), 14 caregivers (82.4%), and 18 youth (90%) participated in Round 2 (i.e. responded to at least one item). The results of the Round 2 survey are displayed in Supplementary File 2, indicating the number and percentage of experts ranking each core component as 8 or 9 (i.e. highly endorsed) for importance or feasibility for each panel.

*Importance* Among the clinicians/administrators, the level of agreement for importance was the greatest for core components 1.6, 4.2, and 4.8 (100.0% highly endorsed), with eight other core components highly endorsed by ≥ 90% of the panel (1.4, 1.7, 2.1, 3.2, 3.3, 4.3, 4.6, and 4.9). Only three core components did not reach ≥ 70% agreement (down from 7 in Round 1; 2.2, 3.1, and 4.11). Among the caregivers, the level of agreement was greatest at 100% for core components 1.4, 4.9, and 4.10, with 10 other core components highly endorsed by ≥ 90% (1.1, 1.6, 3.2, 3.3, 4.2, 4.5–4.7, 5.1 and 5.3). Three core components did not reach ≥ 70% agreement (up from 2 in Round 1; 1.7, 2.1, and 4.11). Among the youth, the level of agreement for importance was the greatest for core component 4.2 (94.4%) and this was the only core component that was highly endorsed by ≥ 90%; four core components did not reach ≥ 70% agreement (down from 7 in Round 1; 2.1, 2.2, 3.1, and 4.1). Across the panels, 20 core components were highly endorsed by ≥ 70% by each group (up from 14 in Round 1), but, mirroring Round 1, none of the core components were endorsed by < 70% in all three panels. There were differences in the core components that were ranked most important and feasible by the panels (see Table 3).

**Table 3:** Top 3 Components Based on Percentage Highly Endorsed per Panel for Feasibility and Importance in Round 2

Clinician		Caregiver		Youth	
Importance	Feasibility	Importance	Feasibility	Importance	Feasibility
1.6^a^	4.8^a^	1.4^a^	3.2	1.3^a^	1.5^a^
4.2^a^	4.9^a^	4.9^a^	4.1	1.4^a^	1.6^a^
4.8^a^	5.3^a^	4.10^a^	4.9	4.2	4.6^a^
				4.10^a^	4.8
				6.1^a^	

^a^Ties in the percentage highly endorsed for top components, resulting in > 3 reported for youth panel

*Feasibility* Similar to Round 1, levels of agreement for feasibility were much lower than for importance. Among the clinicians/administrators, three core components were highly endorsed by > 70% (4.8, 4.9, and 5.3, see Supplementary File 2). For the caregivers, only one core component was endorsed by ≥ 70% (4.9). Among the youth, four core components were endorsed by ≥ 70% (1.5, 1.6, 4.6, and 4.8). None of the core components were endorsed at ≥ 70% in all three panels.

#### Round 2 consensus and final list of core components

Any core component highly endorsed at ≥ 70% on importance or feasibility by at least one panel was retained. This resulted in all 26 core components being retained.

### Final list of transition core components

The final list of transition core components, with all changes made following EAC input and both Delphi Rounds, is available in Table 1 (see column “Final description of core components”).

### Qualitative themes

Three themes were identified from the open-ended qualitative comments provided by experts when asked to elaborate on their reasoning and differences in the rating of the core components in both Rounds. The themes identified were: (1) need for CAMHS and AMHS collaboration; (2) suggestions on how to operationalize core component in practice; and (3) barriers to implementation in practice. A sample of supporting participant quotes for each of these themes are provided in Supplementary File 3. Clinicians and service administrators repeatedly emphasized the need for enhanced communication and active engagement/relationship development between AMHS and CAMHS to increase collaboration between these services. Many participants from all three panels made suggestions on how to implement the core components, such as creating integrated care pathways/plans, ensuring appropriate education and training of involved care providers, and providing readily available online centralized transition resources. A number of barriers to component implementation and feasibility were mentioned in comments across all three expert panels, such as (1) AMHS wait times, (2) challenges posed by differences in CAMHS and AMHS service funding structures, and (3) resources required for training on transitions best practices and for administrative support.

## Discussion

This Delphi study provided the opportunity to refine a list of core components of CAMHS to AMHS transitions with the expertise of youth, caregiver, and clinician/administrators. The initial list of 26 components generated from the scoping review [21] all moved forward to Delphi Round 1 after review by the EAC, with one addition, resulting in 27 components being evaluated. No further components were added after the Delphi rounds (though one was removed), indicating both that the initial scoping review was robust and that the available literature reflects the reported needs and experience of youth, caregivers, and healthcare providers fairly accurately. This method of using an exhaustive literature review to generate the initial survey, rather than having the panels create the list of core components, may allow for Delphi studies to be conducted with reduced burden on the participating experts who are providing their time and energy to assess the survey items. The component that was removed, “Transfer young adult when his/her condition is stable”, was taken out of the list between Rounds 1 and 2. This was because of relatively low endorsement of importance and feasibility as well as comments from all three panels questioning its inclusion due to the complexity inherent to some of the youths’ illnesses and limitations caused by age cut-offs for services. As one caregiver panel member wrote, “stability is often a day to day situation, not an ongoing state in some cases”.

This Delphi study used a multi-panel approach with the goal of balancing the input of health care professionals, youth, and caregivers. Keeping all core components with a high level of endorsement within at least one of the groups, ensured that items deemed highly feasible/important to one group, but not others, would be retained. A number of previous Delphi studies on pediatric to adult health care transitions have used expert panels dominated by, or exclusively made up of, health care professionals with either no or a minority of experts from consumer and/or caregiver groups [22–25, 53, 54]. Among single panel Delphis, most included only a few patient/caregiver members, and as such there is a risk of their rankings carrying less weight than those of health care professionals when attempting to reach a consensus. The balanced panels in this study allow youth and caregivers to be engaged as partners with equal levels of expertise in determining criteria for successful transitions.

Despite the diverse sources of expertise among our panels, there was an overall high level of agreement for importance within and between the expert panels. However, the components with the very highest endorsement for importance within each panel (i.e. each panel’s top three) differed between the youth, caregiver, and clinician/administrator panels. This suggests that youth, caregivers, and clinician/administrators, while agreeing on the importance of the core components overall, may prioritize needs differently when engaged in the transition process. This difference highlights the importance of further engaging patients and families in the co-design of transition interventions based on these core components. Of note, the youth panel had an overall lower level of consensus in their endorsement of core components than either of the other two panels. This may indicate that individual differences between youth accessing care warrant a tailored approach when managing the transition process. This is reflected in existing qualitative research which has found that youth have a high need for flexibility when it comes to the planning and timing of CAMHS to AMHS transitions [26, 28].

Compared to importance, very few core components received a high level of endorsement for feasibility from any of the panels. Experts from all three panels identified that a lack of resources, as well as the siloed administration of child and adult mental health systems, pose significant barriers to implementing the components. Panelists further described in their comments a lack of connection between the two mental health systems, leading to lengthy wait lists for adult services or difficulty finding appropriate adult services, ultimately resulting in long periods of time with no access to mental health care. In many countries, CAMHS and AMHS are completely separate organizations, programs, and/or services [9, 10, 55]. As discussed in the initial literature review [21], separate funding structures and service priorities between CAMHS and AMHS often limits the feasibility of collaboration between child and adult clinicians and programs throughout the transition process.

By the end of Round 2, six components were endorsed for importance by all three panels, and for feasibility by at least one panel. These core components (1.5, 1.6, 4.6, 4.8, 4.9 and 5.3) were focused on role clarity, partnering with youth, and communication of up-to-date information to youth and team members. Given that these components received some endorsement for feasibility (compared to the other core components, which did not receive sufficient endorsement for feasibility by any panels), there may be fewer barriers to their implementation in the current mental health care system. These components were also agreed upon by all panels to be important, indicating that they could represent ideal targets for initial intervention development and evaluation.

Youth have an essential role in contributing to the use of innovative research strategies (such as the Delphi methodology). In this project specifically, youth experts increased the validity of core components and had an active role in the interpretation of the results. In youth mental health research, patient engagement has been increasingly emphasized by funders and in academic literature [41, 44, 56], and recognized as an important pillar of research practice to ensure research success and relevance [42, 57]. Further, through our initial scoping review [21], we identified the need for concensus studies which engaged youth and families as partners in prioritizing components of successful transitions. As a result, this study utilized an integrated knowledge translation process [58] to actively engage youth as equal contributing project partners at all stages of the study. Youth partners led the application of engagement principles across the study, such as ongoing monitoring for youth-friendly language. These contributions ensured that the final list of core components used language that intentionally balanced specificity and flexibility, demonstrating the importance of partnering with youth to best reflect the lived expertise of all stakeholders in study results.

Future work is required to support the operationalization and implementation of these core components into clinical practice. An initial project would be to translate them into a transition checklist specifically created for the context of mental health services, as has been done in chronic or complex health inteventions and settings [59]. This checklist can then guide the design and development of new transitions interventions, the evaluation of existing transition interventions, and the identification of gaps in current interventions and services. This Delphi study has demonstrated the value and feasibility of youth and caregiver engagement. As such, this checklist ought to be co-created with youth and caregivers. Engaging youth and caregivers in the co-design and evaluation of mental health interventions can increase their relevance and impact for youth receiving the services [60], and youth voices should ideally be included at all levels of mental health service provision [61]. Future projects utilizing these components to design, implement, and evaluate transition interventions should continue to engage youth and caregivers to ensure that interventions are appropriate and meaningful for their intended recipients.

## Limitations

While this was a national Canadian study, the panel members were overwhelmingly from Ontario (the most densely populated province in the country), despite multiple attempts to increase recruitment from other provinces and territories. While Canada has a universal public health care system, health care services are administered by provincial and territorial governments. As such variation between provinces in mental health service delivery [62] means the application of these core components in different regions may be limited.

Additonally, the demographic make up of the the youth panel members lacked diversity in terms of gender, race, and diagnosis. For example, while the youth panel reported diversity in cultural groups most identified with, participants overwhelmingly identified as female. Future work should strive for diverse panel members, particularly for sex, gender, race, diagnosis, and primary language, as these youth may have different experiences with transitions in care. Additionally, the use of national list servs for recruitment, while a common method for recruitment in mental health delphi studies [63], may have resulted in less diverse panel membership. Given we have no detail of the demographic make up of list serv members, we have no way of knowing whether the panel members we recruited represented the diversity of the members. This method of recruitment also precludes our ability to determine the response rate of those who received the initial information email to participate versus those who consented to participated. However, the response rate of those who contacted the research team via the informational email versus those who consented was determined and reported in the results.

Another limitation may result from our caregiver panel size. Panel sample sizes of approximately 20 members have been shown to produce stable findings in Delphi studies [32].The caregiver panel was smaller than this at 17 participants and this group experienced a greater level of attrition than the other panels between the two rounds. Further, as part of maintaining participants’ confidentiality, their last names were not requested on the survey, leading to the possibility that there were caregiver-youth dyads participating in the survey. Inclusion of dyads in the survey responses may have limited the diversity of the panels and could have the potential to bias the results based on specific shared experiences within these dyads.

Finally, this study resulted in a very low level of concensus for feasibility of the core components. Previous work using the Delphi methodology has found that panelists report that assessment of ‘feasibility’ can be challenging [64], which, along with the low levels of endorsement found in the current study, indicates that this may be a concept that is not sufficiently concrete or specific to ask experts to rate in future Delphi studies.

## Conclusion

This Delphi study applied the principles of patient-oriented research to refine a list of core components for successful transitions between child and adult mental health services. Authentic engagement of youth and families as equal experts alongside healthcare professionals has resulted in a list of core components which reflect the identified needs and lived experience of service users as well as providers. These 26 core components should be used in the design and evaluation of programs or interventions to improve transitions from CAMHS to AMHS. Ultimately, the development and evaluation of transition interventions are needed to promote safe and seamless transitions and improve outcomes for youth requiring ongoing mental health care.

## Supplementary Information

Below is the link to the electronic supplementary material.

Supplementary file1 (DOCX 23 kb)

Supplementary file2 (DOCX 23 kb)

Supplementary file3 (DOCX 14 kb)

## Data Availability

The data are available from the authors upon reasonable request—subject to ethical permissions and participant consent.
