# Supplementary material for: Prioritizing core components of successful transitions from child to adult mental health care: a national Delphi survey with youth, caregivers, and health professionals
Source: Eur Child Adolesc Psychiatry. 2021 Jun 5;31(11):1739–52. doi: 10.1007/s00787-021-01806-6 (PMC9666300; doi:10.1007/s00787-021-01806-6)
Supplement: Supplementary file 1 — Supplementary file1 (DOCX 23 kb) [file 787_2021_1806_MOESM1_ESM.docx]

**Supplementary File 2.** Percentage of panel experts rating components at 8 or 9 for importance/feasibility in Round 2

^a^ Highlighted cells indicate a rating of 8 or 9 (i.e. high endorsement) for feasibility or importance by $\geq$70% of the expert panel

| Component | Importance^a^ | | | | | | Feasibility^a^ | | | | | |
| --- | --- | --- | --- | --- | --- | --- | --- | --- | --- | --- | --- | --- |
|  | **Clinician (n=19)** | | **Caregiver (n=14)** | | **Youth (n=18)** | | **Clinician (n=19)** | | **Caregiver (n=14)** | | **Youth (n=18)** | |
|  | Valid N | % rated  8 or 9 | Valid N | % rated  8 or 9 | Valid N | % rated  8 or 9 | Valid N | % rated  8 or 9 | Valid N | % rated  8 or 9 | Valid N | % rated  8 or 9 |
| **1.1** | 18 | 88.9 | 14 | 92.9 | 18 | 77.8 | 18 | 16.7 | 14 | 35.7 | 18 | 38.9 |
| **1.2** | 18 | 77.8 | 14 | 71.4 | 18 | 72.2 | 18 | 55.6 | 14 | 42.9 | 18 | 50.0 |
| **1.3** | 18 | 88.9 | 14 | 85.7 | 18 | 88.9 | 18 | 33.3 | 14 | 42.9 | 18 | 38.9 |
| **1.4** | 18 | 94.4 | 14 | 100.0 | 18 | 88.9 | 18 | 27.8 | 13 | 38.5 | 18 | 66.7 |
| **1.5** | 18 | 88.9 | 12 | 75.0 | 18 | 77.8 | 18 | 27.8 | 13 | 46.2 | 18 | 72.2 |
| **1.6** | 18 | 100.0 | 14 | 92.9 | 18 | 83.3 | 18 | 44.4 | 14 | 42.9 | 18 | 72.2 |
| **1.7** | 18 | 94.4 | 14 | 64.3 | 18 | 72.2 | 18 | 50.0 | 14 | 50.0 | 18 | 55.6 |
| **2.1** | 18 | 94.4 | 14 | 64.3 | 18 | 61.1 | 18 | 61.1 | 14 | 57.1 | 18 | 27.8 |
| **2.2** | 18 | 61.1 | 14 | 78.6 | 18 | 66.7 | 17 | 35.3 | 14 | 57.1 | 17 | 52.9 |
| **3.1** | 19 | 57.9 | 14 | 78.6 | 18 | 66.7 | 17 | 41.2 | 14 | 35.7 | 18 | 50.0 |
| **3.2** | 18 | 94.4 | 13 | 92.3 | 18 | 83.3 | 18 | 44.4 | 13 | 69.2 | 16 | 68.8 |
| **3.3** | 18 | 94.4 | 13 | 92.3 | 18 | 83.3 | 18 | 55.6 | 14 | 42.9 | 18 | 44.4 |
| **4.1** | 17 | 82.4 | 14 | 85.7 | 18 | 61.1 | 17 | 52.9 | 14 | 64.3 | 18 | 50.0 |
| **4.2** | 18 | 100.0 | 13 | 92.3 | 18 | 94.4 | 18 | 22.2 | 14 | 50.0 | 18 | 55.6 |
| **4.3** | 18 | 94.4 | 14 | 78.6 | 18 | 77.8 | 17 | 64.7 | 14 | 57.1 | 18 | 66.7 |
| **4.4** | 18 | 83.3 | 14 | 78.6 | 18 | 77.8 | 18 | 33.3 | 14 | 28.6 | 18 | 38.9 |
| **4.5** | 17 | 76.5 | 14 | 92.9 | 18 | 83.3 | 19 | 31.6 | 14 | 42.9 | 18 | 55.6 |
| **4.6** | 17 | 94.1 | 14 | 92.9 | 18 | 83.3 | 17 | 41.2 | 14 | 42.9 | 18 | 72.2 |
| **4.7** | 18 | 83.3 | 14 | 92.9 | 18 | 77.8 | 18 | 27.8 | 14 | 35.7 | 18 | 38.9 |
| **4.8** | 18 | 100.0 | 14 | 78.6 | 18 | 77.8 | 18 | 72.2 | 14 | 57.1 | 18 | 77.8 |
| **4.9** | 18 | 94.4 | 14 | 100.0 | 18 | 77.8 | 18 | 72.2 | 14 | 71.4 | 18 | 61.1 |
| **4.10** | 18 | 88.9 | 14 | 100.0 | 18 | 88.9 | 18 | 55.6 | 14 | 57.1 | 18 | 66.7 |
| **4.11** | 18 | 50.0 | 14 | 64.3 | 18 | 77.8 | 18 | 16.7 | 14 | 28.6 | 18 | 55.6 |
| **5.1** | 18 | 72.2 | 14 | 92.9 | 18 | 83.3 | 19 | 31.6 | 14 | 42.9 | 18 | 38.9 |
| **5.2** | *N/A removed after Round 1* | | | | | | | | | | | |
| **5.3** | 18 | 88.9 | 13 | 92.3 | 18 | 83.3 | 18 | 72.2 | 13 | 46.2 | 18 | 66.7 |
| **6.1** | 17 | 70.6 | 14 | 85.7 | 18 | 88.9 | 18 | 22.2 | 13 | 61.5 | 18 | 61.1 |
