# Supplementary material for: Prioritizing core components of successful transitions from child to adult mental health care: a national Delphi survey with youth, caregivers, and health professionals
Source: Eur Child Adolesc Psychiatry. 2021 Jun 5;31(11):1739–52. doi: 10.1007/s00787-021-01806-6 (PMC9666300; doi:10.1007/s00787-021-01806-6)
Supplement: Supplementary file 2 — Supplementary file2 (DOCX 23 kb) [file 787_2021_1806_MOESM2_ESM.docx]

**Supplementary File 3.** Themes and exemplar quotes from panel experts in Round 1 and 2.

| **Exemplar quotes from panel experts** | |
| --- | --- |
| **Theme 1:**  **CAMHS & AMHS collaboration** | “If AMHS run parallel to CAMHS during transition, sharing documents is less critical, as they are obtaining information/exposure to the youth directly.”  *Caregiver (Round 1, Component 5.3)* |
|  | “There needs to be real collaboration between the two for this to work. I feel some groundwork will need to be done around changing cultural thinking about seamless services for youth, especially in the adult side of services.”  *Clinician (Round 1, Component 1.3)* |
|  | “… I see the need for ministry level expectations to be shared that provide a clear and fully informed expectation of transition service improvement that does not allow for organizational influence to be greater than the required outcome for improved transition.” *Administrator (Round 2, Component 1.2)* |
| **Theme 2: Recommendations on how to operationalize the core components** | “As well, I think it should be a policy made by transitional aged youth, as opposed to input by youth.”  *Youth (Round 1, Component 1.2)* |
|  | “In addition to criteria, there may need to be some room for understanding special circumstances so the system is not too rigid.”  *Caregiver (Round 1, Component 2.1)* |
|  | “I don't think additional paperwork is useful. Instead, I suggest a clearly laid-out protocol, that the clinician follows (and charts in the usual progress notes)”  *Clinician (Round 2, Component 4.5)* |
|  | “Requires high quality systems to support this so it becomes part of care not an addition to care. Otherwise affects capacity to provide services.”  *Administrator (Round 1, Component 4.8)* |
| **Theme 3:**  **Barriers to implementation** | “This seems like a good idea, however, it seems highly unfeasible as this would probably take a lot of time on behalf of both the AMHS provider and the CAMHS clinician. It may take away from their time being able to provide services to those who need it”  *Youth (Round 1, Component 4.7)* |
|  | “optimal timing is great, but reality is waitlists and age/birthday dictate the schedule more than the optimal schedule ever would/could”  *Caregiver (Round 1, Component 5.1)* |
|  | “Until the referral has been made and accepted and the youth has been assessed (which cannot be done before a certain age) there IS no adult clinician to collaborate with” *Clinician, Round 2, Component 4.7* |
|  | “It would take a significant amount of resources - time, training, information to ensure that staff are trained. My experience in the CYMH sector is that there are very few resources and staff are already stretched to the maximum”  *Administrator (Round 1, Component 1.5)* |
