# Supplementary material for: Prioritizing core components of successful transitions from child to adult mental health care: a national Delphi survey with youth, caregivers, and health professionals
Source: Eur Child Adolesc Psychiatry. 2021 Jun 5;31(11):1739–52. doi: 10.1007/s00787-021-01806-6 (PMC9666300; doi:10.1007/s00787-021-01806-6)
Supplement: Supplementary file 3 — Supplementary file3 (DOCX 14 kb) [file 787_2021_1806_MOESM3_ESM.docx]

| Component | Importance^a^ | | | | | | Feasibility^a^ | | | | | |
| --- | --- | --- | --- | --- | --- | --- | --- | --- | --- | --- | --- | --- |
|  | **Clinician (n=21)** | | **Caregiver (n=17)** | | **Youth (n=20)** | | **Clinician (n=21)** | | **Caregiver (n=17)** | | **Youth (n=20)** | |
|  | Valid N | % rated  8 or 9 | Valid N | % rated  8 or 9 | Valid N | % rated  8 or 9 | Valid N | % rated  8 or 9 | Valid N | % rated  8 or 9 | Valid N | % rated  8 or 9 |
| **1.1** | 21 | 71.4 | 17 | 76.5 | 19 | 68.4 | 21 | 28.6 | 17 | 41.2 | 19 | 31.6 |
| **1.2** | 20 | 70.0 | 17 | 82.4 | 20 | 70.0 | 20 | 45.0 | 17 | 58.8 | 20 | 30.0 |
| **1.3** | 20 | 80.0 | 15 | 86.7 | 19 | 68.4 | 20 | 35.0 | 15 | 53.3 | 19 | 26.3 |
| **1.4** | 20 | 95.0 | 16 | 87.5 | 19 | 89.5 | 20 | 25.0 | 16 | 56.3 | 19 | 63.2 |
| **1.5** | 20 | 90.0 | 15 | 86.7 | 19 | 78.9 | 20 | 50.0 | 15 | 66.7 | 19 | 47.4 |
| **1.6** | 20 | 100.0 | 16 | 81.3 | 19 | 94.7 | 20 | 45.0 | 15 | 53.3 | 19 | 63.2 |
| **1.7** | 20 | 90.0 | 16 | 75.0 | 17 | 82.4 | 20 | 55.0 | 16 | 62.5 | 17 | 47.1 |
| **2.1** | 20 | 85.0 | 15 | 73.3 | 18 | 66.7 | 20 | 50.0 | 14 | 57.1 | 18 | 38.9 |
| **2.2** | 20 | 60.0 | 15 | 53.3 | 18 | 72.2 | 20 | 55.0 | 14 | 57.1 | 18 | 55.6 |
| **3.1** | 20 | 60.0 | 15 | 80.0 | 19 | 73.7 | 20 | 30.0 | 15 | 53.3 | 19 | 68.4 |
| **3.2** | 20 | 90.0 | 15 | 86.7 | 19 | 78.9 | 20 | 55.0 | 15 | 73.3 | 19 | 68.4 |
| **3.3** | 20 | 90.0 | 15 | 86.7 | 19 | 89.5 | 19 | 31.6 | 15 | 66.7 | 19 | 47.4 |
| **4.1** | 19 | 89.5 | 14 | 85.7 | 17 | 64.7 | 20 | 60.0 | 14 | 71.4 | 17 | 41.2 |
| **4.2** | 20 | 95.0 | 14 | 85.7 | 18 | 94.4 | 20 | 40.0 | 14 | 64.3 | 17 | 70.6 |
| **4.3** | 20 | 95.0 | 14 | 64.3 | 17 | 88.2 | 20 | 70.0 | 14 | 50.0 | 17 | 64.7 |
| **4.4** | 20 | 80.0 | 13 | 84.6 | 18 | 66.7 | 20 | 30.0 | 14 | 64.3 | 18 | 38.9 |
| **4.5** | 19 | 68.4 | 14 | 85.7 | 18 | 83.3 | 19 | 47.4 | 13 | 61.5 | 18 | 50.0 |
| **4.6** | 20 | 85.0 | 14 | 92.9 | 18 | 66.7 | 20 | 65.0 | 13 | 69.2 | 18 | 61.1 |
| **4.7** | 20 | 70.0 | 14 | 92.9 | 18 | 72.2 | 19 | 10.5 | 14 | 64.3 | 18 | 27.8 |
| **4.8** | 19 | 94.7 | 14 | 78.6 | 18 | 72.2 | 20 | 70.0 | 14 | 71.4 | 18 | 55.6 |
| **4.9** | 20 | 95.0 | 14 | 71.4 | 18 | 72.2 | 20 | 70.0 | 14 | 78.6 | 18 | 77.8 |
| **4.10** | 20 | 90.0 | 14 | 78.6 | 17 | 88.2 | 20 | 65.0 | 13 | 76.9 | 18 | 66.7 |
| **4.11** | 20 | 50.0 | 14 | 85.7 | 18 | 83.3 | 20 | 15.0 | 14 | 42.9 | 18 | 38.9 |
| **5.1** | 20 | 70.0 | 14 | 78.6 | 17 | 76.5 | 20 | 35.0 | 14 | 57.1 | 17 | 52.9 |
| **5.2** | 20 | 45.0 | 13 | 76.9 | 17 | 64.7 | 20 | 20.0 | 12 | 25.0 | 17 | 29.4 |
| **5.3** | 20 | 80.0 | 14 | 78.6 | 17 | 82.4 | 20 | 65.0 | 14 | 57.1 | 17 | 64.7 |
| **6.1** | 20 | 65.0 | 14 | 85.7 | 18 | 72.2 | 20 | 40.0 | 14 | 71.4 | 18 | 33.3 |

**Supplementary File 1.** Percentage of panel experts rating components at 8 or 9 for importance/feasibility in Round 1

^a^ Highlighted cells indicate a rating of 8 or 9 (i.e. high endorsement) for feasibility or importance by $\geq$70% of the expert panel
